# Clinical outcomes of conventional versus extended mesenteric resection in limited ileo-colonic Crohn’s disease: a systematic review and meta-analysis

**DOI:** 10.1007/s00384-025-04937-3

**Published:** 2025-06-18

**Authors:** Omar E. S. Mostafa, Shafquat Zaman, Maymunah Malik, Prajeesh Kumar, Lalit Kumar, Akinfemi Akingboye, Diwakar Sarma, Rajeev Peravali

**Affiliations:** 1 Department of General and Colorectal Surgery, Dudley Group NHS Foundation Trust, Dudley, West Midlands, UK; 2 Department of General and Colorectal Surgery, University Hospital of Derby and Burton NHS Foundation Trust, Burton On Trent, Staffordshire UK; 3 School of Medical Sciences, College of Medicine and Health, University of Birmingham, Edgbaston, Birmingham, UK; 4 Aston Medical School, College of Health and Life Sciences, Aston University, Birmingham, UK; 5 Department of General and Colorectal Surgery, Sandwell and West Birmingham Hospitals NHS Foundation Trust, Birmingham, UK

**Keywords:** Limited, Conventional, Extended, Mesentery, Crohn’s disease, Outcomes

## Abstract

**Background:**

The role of intestinal mesentery and the extent of its resection as a determinant of outcomes post-bowel resection in Crohn’s disease (CD) remains a subject of debate. We evaluated outcomes of conventional mesenteric resection (CMR) and compared it with extended mesenteric resection (EMR) in patients undergoing ileo-colic excision for limited ileo-colonic CD.

**Methods:**

A comprehensive search was conducted in accordance with PRISMA guidelines using Medline, Embase, PubMed, and Cochrane databases. Comparative studies of patients with limited ileo-colonic CD undergoing CMR and EMR for ileo-colic resection were included. Studies comparing anastomotic techniques, single-arm, case reports/series, study protocols and editorials were excluded. Primary outcomes were disease recurrence and re-operation. Secondary outcomes included post-operative complications, intra-operative blood loss, length of stay, total operative time and re-admission rate. Meta-analysis was performed using Cochrane RevMan Web on outcomes reported by two or more studies. Combined overall effect sizes were calculated using random-effects model and the Newcastle–Ottawa Scale and Cochrane risk-of-bias tools were used to assess bias.

**Results:**

Five studies met our inclusion criteria (four retrospective cohort studies; one randomised controlled trial (RCT)) with a total of 4,358 patients (EMR: 993 vs. CMR: 3,365). No statistical difference was observed across any of the analysed outcomes: disease recurrence [OR: 0.49 CI 0.21—1.16, P = 0.10], re-operation [OR: 0.33 CI 0.06—1.65, P = 0.17], intra-operative blood loss [MD: -18.71 CI -76.65—39.23, P = 0.53], anastomotic leak [OR: 0.98 CI 0.34—2.82, P = 0.97], length of stay [MD: -0.06 CI -0.59—0.48, P = 0.83], post-operative morbidity [OR: 1.01 CI 0.82—1.24, P = 0.95], blood transfusion [OR: 1.16 CI 0.84—1.59, P = 0.36], Clavien-Dindo III + complications [OR: 0.83 CI 0.5—1.38, P = 0.47], post-operative ileus [OR: 0.97 CI 0.27—3.50, P = 0.96], intra-abdominal bleeding [OR: 0.85 CI 0.22—3.26, P = 0.81], re-admission [OR: 0.65 CI 0.24—1.78, P = 0.40], surgical site infection [OR: 1.00 CI 0.77—1.30, P = 0.99], post-operative adjuvant or prophylactic therapy [OR: 0.90 CI 0.54—1.51, P = 0.69] and total operative time [MD: 0.38 CI -4.42—5.19, P = 0.88].

**Conclusion:**

Performing EMR during ileo-colic resection for patients with limited ileo-colonic CD does not seem to confer any additional benefit to conventional (limited resection) approaches. Robust, well-designed, large-scale RCTs are needed to better compare these techniques and demonstrate superiority in clinical outcomes.

## Introduction

An estimated 50% of patients with Crohn’s disease (CD) may develop recurrent disease following their index surgical intervention [1]. The involvement of bowel mesentery (composed of adipose, nervous, immune, lymphatic and stromal tissue) is a hallmark of CD pathogenesis [2]. Microscopically, disease recurrence (in most instances) has been observed on the mesenteric border of the bowel, suggesting benefit in excising the mesentery to reduce post-operative recurrence [3].

However, there is a paucity of literature and no consensus to date on the optimal surgical technique to reduce the risk of CD recurrence. The role of the mesentery and the extent of its excision (conventional/close wall (CMR) vs. extended (EMR)) remains controversial.

Current European Crohn’s and Colitis Organisation (ECCO) guidelines support a mesenteric-sparing approach [4]. The recently published international randomised controlled trial (RCT)—effect of mesenteric sparing or extended resection in primary ileocolic resection for Crohn's disease on postoperative endoscopic recurrence (SPICY), reported no difference/non-inferiority in endoscopic recurrence or post-operative outcomes between limited/conventional and EMR [5]. However, some observational data has suggested that EMR may be associated with longer recurrence-free periods of active disease and a lower incidence of post-operative complications compared with the conventional approach [6, 7].

With the exception of the SPICY trial data, there is little in the way of high-level published evidence in the literature comparing outcomes between patients undergoing CMR and EMR. The on-going MEsenteric Excision and Kono-S Anastomosis Trial** (**MEErKAT)—a UK-wide multi-centre study examining outcomes associated with mesenteric excision and/or Kono-S anastomosis in patients undergoing ileo-colic resection, and its results are eagerly awaited [8]. Additionally, Li et al. [9] have also published a study protocol for an international, multi-centre RCT to test whether meso-colic excision to CD reduces post-operative disease recurrence and morbidity.

In view of conflicting evidence, we performed a systematic review and meta-analysis of the available literature and compared CMR vs. EMR in patients with limited ileo-colonic CD undergoing ileo-colic resection. We compared disease recurrence, post-operative morbidity and clinical outcomes between the two techniques.

## Materials and methods

The systematic review and meta-analysis was conducted in accordance with the Cochrane Handbook for Systematic Reviews and Meta-Analyses [10] and the Preferred Reporting Items for Systematic Reviews and Meta-Analyses (PRISMA) guidelines [11] and Assessing the Methodological Quality of Systematic Reviews (AMSTAR 2) guidelines [12]. The protocol was registered on the international prospective register of systematic reviews (PROSPERO registration number: CRD42024589678, available from: https://www.crd.york.ac.uk/PROSPERO/view/CRD42024589678). No prior ethical approval was required to conduct this review.

### Search strategy and study selection

We comprehensively searched the literature using four databases: Medline, Embase, Cochrane and PubMed. These electronic data sources were searched from inception to 12th September 2024 for all comparative studies of adult patients (> 18 years) with limited ileo-colonic CD needing ileocolonic resection, undergoing either EMR or CMR. Our intervention group was EMR, and the comparator was CMR. Key words included “*mesenteric resection*” OR *“mesenteric excision”* OR *“mesentery sparing”* AND *“limited”* OR *“conventional”* OR *“extended”* AND *“Crohns Disease”* OR *“ileocolic”* OR *“ileocolonic”*. The term ‘extended’ was defined in line with previously published studies; the extent of resection of the mesentery to its origin from the mesenteric vessels, rather than the traditional concept which refers to ‘length’ of bowel excised. Our primary outcomes were disease recurrence (either endoscopically or surgically diagnosed) and re-operation. Our secondary outcomes were post-operative complications (paralytic ileus, anastomotic leak, intra-abdominal haemorrhage, wound infections, need for blood transfusion, blood loss), length of stay (LOS), re-admission rates and post-operative adjuvant therapy for CD. Two independent authors conducted searches, title screening and full-text screening before inclusion. A third author resolved any discrepancies.

### Inclusion and exclusion criteria

**Fig. 1:**
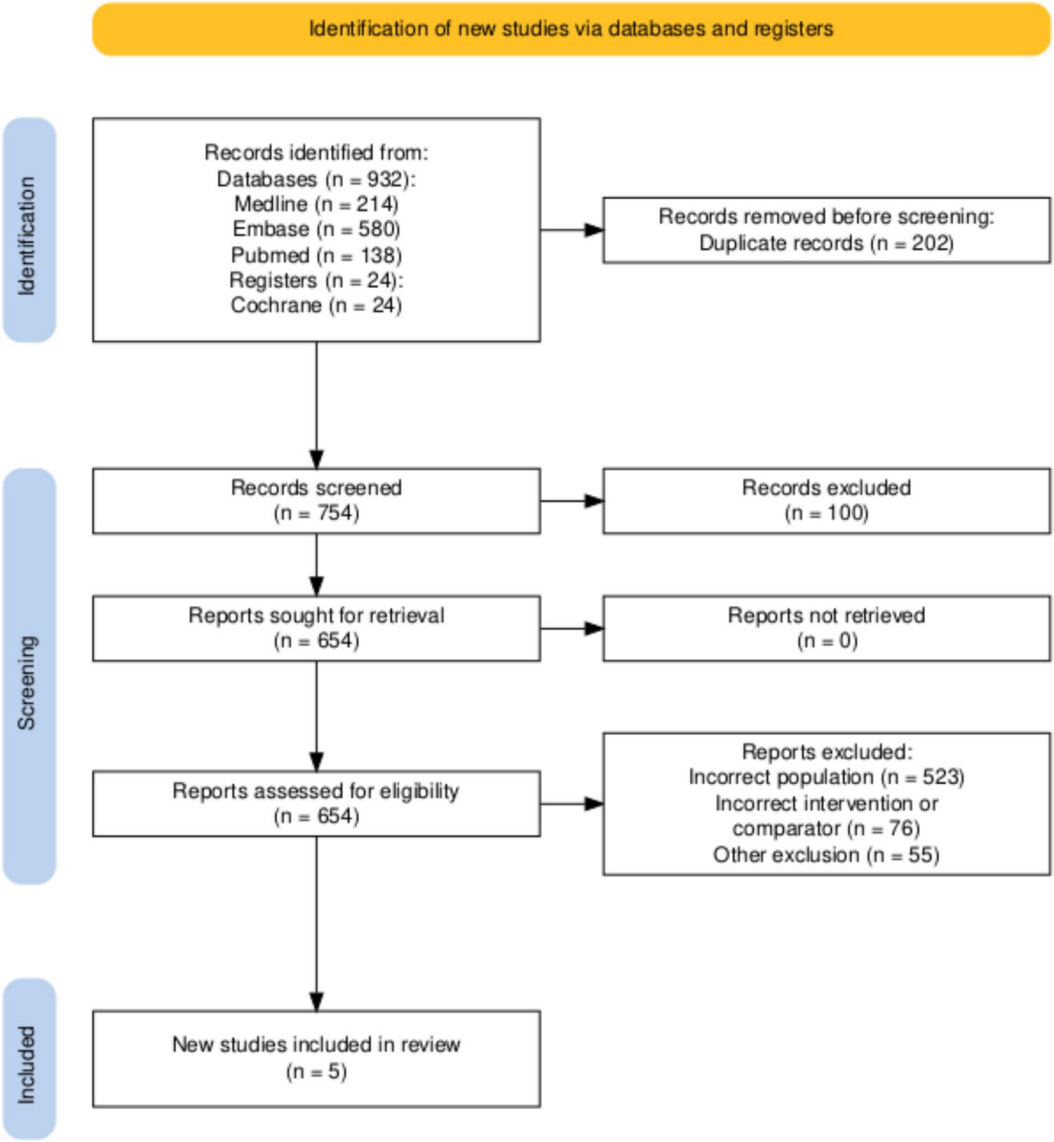
PRISMA Flowchart

Only comparative observational or experimental studies of EMR and CMR were included. We specifically excluded studies comparing various anastomotic techniques, published protocols for clinical trials, single-arm studies, case reports and series, conference abstracts, online posters, reviews and editorials. No language restrictions were applied Fig. 1.

### Data extraction

A Microsoft Excel (Microsoft, v13.5) spreadsheet was formulated for data extraction [13]. Data extracted was categorised for both CMR and EMR based on study-related data *(name of author, country, title, population in each arm, intervention, inclusion/exclusion criteria, outcomes)*, baseline demographics *(average age, male gender, smoking, average BMI, hypertension, diabetes, chronic kidney disease, presence of ileocolic CD, presence of penetrating CD)* and clinical outcomes (*disease recurrence, blood loss, operative time, LOS, blood transfusion, time to return of bowel movement, re-admission, re-operation, postoperative morbidity, Clavien-Dindo 3* + *complications, Surgical Site Infections (SSI), intra-abdominal abscess formation, intra-abdominal bleeding, paralytic ileus, gastrointestinal dysfunction, and post-operative adjuvant therapy)*. These are summarised in Tables 1, 2 and 3, respectively.

**Table 1:** Study related demographics

Author	Year	Country	Journal	Type of study	Population	Interventions	Exclusion Criteria	Outcome(s) Measured
Coffey et al. [6]	2018	Ireland	Journal of Crohn's and Colitis	Retrospective, Cohort	Patients with ileocolic CD between Jan 2004 and April 2010, and August 2010	Mesentery spared (Group A 30/64), Mesentery removed (Group B 34/64)	N/A	Surgical recurrence, disease activity, microscopic appearances
Zhu et al. [7]	2021	China	Clinical and Translational Gastroenterology	Retrospective, Cohort	Adult patients with CC who underwent colorectal resection between January 2000 and December 2018	Limited Mesenteric Excision (60/126), Extended Mesenteric Excision (66/126)	(i) age, 18 years, (ii) history of colorectal resection, (iii) concomitant jejunal or ileal resection, (iv) abdominoperineal resections for perianal disease, (v) fecal diversion without colorectal resection, or (vi) surgery for dysplasia and colorectal cancer complicating CD	Post-operative outcomes (surgical recurrence) and early post-operative complications
Mineccia et al. [16]	2022	Italy	Journal of Clinical Medicine	Retrospective, Cohort	Consecutive, unselected patients with a single location of CD localized to the terminal ileum, operated on between January 2009 and December 2019	Mesentery removal (Group A 204/326), Mesentery spared (Group B 122/326)	Patients with proximal jejuno-ileal or colonic locations were excluded, even if those locations were not suitable for surgery	Duration of surgery, Clavien–Dindo complications, hospitalization, 90-day readmission, endoscopic and ultrasonographic follow-up evaluation within one year from surgery, post-operative adjuvant treatment, and long-term surgical recurrence
Abdulkarim et al. [15]	2023	Canada	International Journal of Colorectal Disease	Retrospective, Cohort	Patients with CD undergoing segmental colectomy between 2014 and 2019	Extended Mesenteric Resection (622/3709), Limited Mesenteric Resection (3087/3709)	Patient with a concomitant diagnosis of colorectal cancer or who received chemotherapy within 90 days were excluded	NSQIP Major morbidity, SSI, dehiscence, sepsis, MI, cardiac arrest, ARF, Pneumonia, DVT, UTI, unplanned intubation and re-operation, abdominal complications, post-op bleeding
van der Does de Willebois et al. [5]	2024	Netherlands, Italy	Lancet	Randomised Controlled Trial	Adult patients with Crohns disease previously confirmed by endoscopy in the terminal ileum or ileocolic region with a recent update (within the last 3 months) of imaging (ultrasound, MRI, or CT enterography)	Extended Mesenteric Resection (67/133), Mesenteric Sparing Resection (66/133)	Previous ileocolic resection, clinically significant medical conditions within 6 months before the operation Previous diagnosis of cancer with influence on the patient’s prognosis, emergent operation, pregnancy or breastfeeding, and inability to comply with postoperative assessments	Endoscopic recurrence 6 months postoperatively, severity of post operative recurrence, operative time, intraoperative bleeding, blood loss, conversion rate, post op LOS, anastamotic leakage, histopathological data, use of post op Crohns meds, adverse events

*CC* Crohns Colitis, *CD* Crohns Disease, *MRI* Magnetic Resonance Imaging, *CT* Computerised Tomography, *N/A* Not applicable, *NSQIP* National Surgical Quality Improvement Programme, *SSI* Surgical Site Infection, *ARF* Acute Renal Failure, *MI* Myocardial infarction, *DVT* Deep Vein Thrombosis, *UTI* Urinary Tract Infection, *LOS* Length of stay

**Table 2:** Patient demographics of populations included per study

Author	Total patients (n)	Extended Resection (n)	Limited Resection (n)	Mean Age (Y)	Male Gender (%)	Smoking (%)	Average BMI (kg/m^2^)	HTN (%)	Diabetes (%)	CKD (%)	Ileocolic Disease (%)	Penetrating Disease (%)
Coffey et al. [6]	64	34	30	35.9 vs 37.7	41% vs 47%	53% vs 47%	N/A	N/A	N/A	N/A	18% vs 17%	35% vs 27%
Zhu et al. [7]	126	66	60	25.23 vs 26.85	63.6% vs 78.3%	7.6% vs 15%	18.04 vs 18.48	N/A	N/A	N/A	37.9% vs 46.7%	36.4% vs 66.7%
Mineccia et al. [16]	326	204	122	40.5 vs 40.7	59.3% vs 57.4%	36.8% vs 28.7%	N/A	N/A	N/A	N/A	N/A	67.2% vs 67.2%
Abdulkarim et al. [15]	3709	622	3087	41 vs 42.3	48% vs 45.6%	19.8% vs 22.6%	25.3 vs 25.9	16.7% vs 16.8%	4.7% vs 3.4%	0.2% vs 0.1%	N/A	N/A
van der Does de Willebois et al. [5]	133	67	66	36 vs 37	43% vs 42%	22% vs 24%	N/A	N/A	N/A	N/A	34% vs 39%	25% vs 33%

Results comparing extended resection against limited resection

*BMI* Body Mass Index, *HTN* Hypertension, *CKD* Chronic Kidney Disease, *N/A* Not Applicable, *Y* Years

**Table 3:** Summary of outcomes per study

Author	Patients (n)	Disease Recurrence, n (%)	Mean blood loss (mls ± SD)	Operative Time, n (%)	LOS (days, SD or range)	Blood transfusion, n (%)	Readmission, n (%)	Reoperation, n (%)	Postoperative Morbidity, n (%)	C-D 3 +, n (%)	SSI, n (%)	Leak, n (%)	Ileus, n (%)	Intraabdominal bleeding, n (%)	Adjuvant therapy, n (%)
Coffey et al. [6]	Extended: 34, Limited: 30	1 (2.9%) vs 12 (40%)	N/A	N/A	N/A	N/A	N/A	1 (2.9%) vs 12 (40%)	N/A	N/A	N/A	N/A	N/A	N/A	7 (21%) vs 6 (20%)
Zhu et al. [7]	Extended: 66, Limited: 60	7 (10.6%) vs 18 (30%)	**83.10 ± 56.19 vs 132.25 ± 85.53** **[P = 0.002]**	N/A	10.49 ± 5.17 vs 12.64 ± 8.57	6 (9.1%) vs 4 (6.7%)	2 (3%) vs 2 (3.3%)	7 (10.6%) vs 18 (30%)	17 (25.8%) vs 14 (23.3%)	N/A	3 (17.6%) vs 4 (28.6%)	2 (11.8%) vs 5 (35.7%)	2 (11.8%) vs 0 (0%)	0 (0%) vs 2 (14.3%)	40 (60.6%) vs 30 (50%)
Mineccia et al. [16]	Extended: 204, Limited: 122	91 (44.6%) vs 57 (46.7%)	N/A	150 ± 54 vs 146 ± 55	8.5 ± 5 vs 9 ± 4	N/A	6 (3%) vs 6 (4.9%)	N/A	48 (23.5%) vs 33 (27%)	37 (18.1%) vs 28 (23%)	N/A	N/A	N/A	N/A	117 (65%) vs 87 (59.5%)
Abdulkarim et al. [15]	Extended: 622, Limited: 3087	N/A	N/A	170 ± 78.3 vs 166.5 ± 73.75	7.07 ± 8.95 vs 7.02 ± 7.60	48 (7.72%) vs 210 (6.8%)	N/A	31 (4.98%) vs 126 (4.08%)	91 (14.63%) vs 442 (14.32%)	N/A	73 (11.74%) vs 359 (11.63%)	22 (3.54%) vs 118 (3.82%)	74 (11.90%) vs 477 (15.45%)	48 (7.72%) vs 210 (6.8%)	N/A
van der Does de Willebois et al. [5]	Extended: 66, Limited: 65	28 (42%) vs 28 (43%)	50 vs 50	170 vs 167	5 (4–7) vs 5 (3–7)	N/A	N/A	N/A	7 (11%) vs 5 (8%)	7 (11%) vs 5 (8%)	N/A	5 (8%) vs 1 (2%)	N/A	N/A	34 (52%) vs 34 (52%)

Results in bold indicate a statistically significant reported outcome

Results are reported as number of patients (n) with percentage (%) of total comparing extended versus limited resection

*LOS* Length of stay, *SD* Standard Deviation, C-D 3 + : Clavien Dindo 3 +, *SSI* Surgical Site Infection, *N/A* Not Applicable

### Quality assessment

**Fig. 2:**
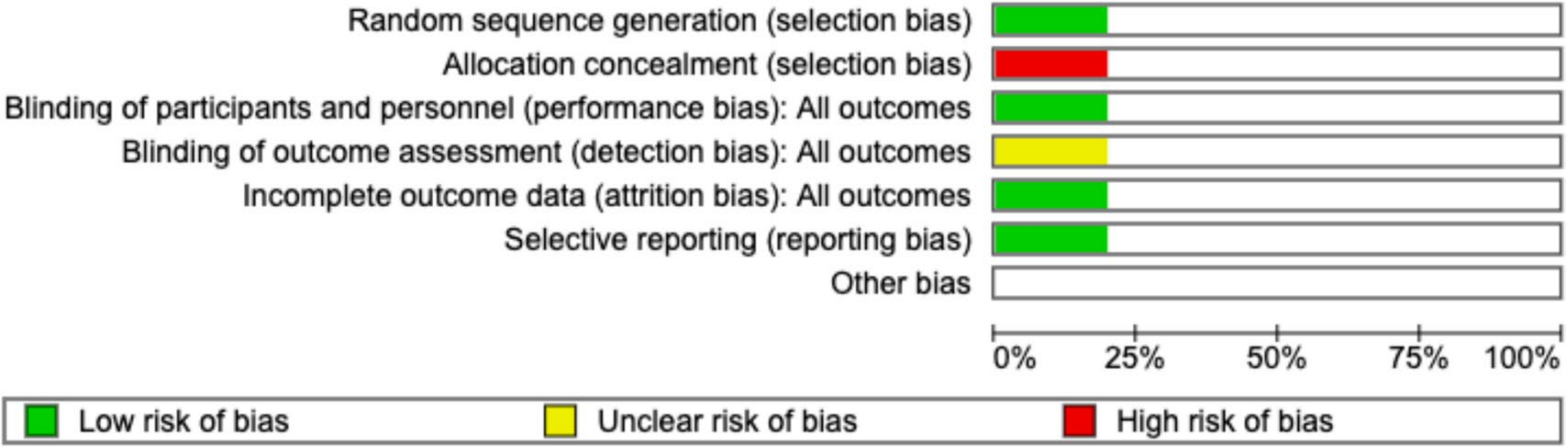
Risk of Bias (RoB) Graph for *van der Does de Willebois *et al.,* 2024* [5]

For observational studies, the Newcastle–Ottawa Score (NOS) was used [14]; an-asterisk based points system assessing the selection, comparability and ascertainment of exposure in the selected cohorts with a score of 6 or below deeming the study to be at high risk of bias [Table 4]. For experimental studies, the Cochrane Risk of Bias (RoB) tool [10] was used to assess studies based on random sequence generation, allocation concealment, blinding of participants and personnel, blinding of outcome assessment, incomplete outcome data, selective reporting, and other sources of bias [Fig. 2].

**Table 4:** NOS for the risk of bias and quality assessment of NRSs

Author	Year	Selection				Comparability	Outcome			Total score	Total Quality
		Representativeness of the Exposed Cohort	Selection of the Non-Exposed Cohort	Ascertainment of Exposure	Demonstration That Outcome of Interest Was Not Present at Start of Study	Comparability of Cohorts on the Basis of the Design or Analysis	Assessment of Outcome	Was Follow-Up Long Enough for Outcomes to Occur	Adequacy of Follow Up of Cohorts		
Coffey et al. [6]	2018	*	*	*	*		*	*	*	7	Good
Zhu et al. [7]	2021	*	*	*		*	*	*	*	7	Good
Mineccia et al. [16]	2022	*	*	*		*	*	*	*	7	Good
Abdulkarim et al. [15]	2023	*	*	*	*	*	*	*	*	8	Good

*NOS* Newcastle–Ottawa scale, *NRS* non-randomized study. One Asterisk denotes a point. A score < 6 indicates poor quality

### Statistical analysis

Statistical and meta-analysis were performed using Cochrane Review Manager (RevMan) [Computer Programme], version 7.2 (Cochrane Collaboration, 2024) based on a random-effects model using the Mantel–Haenszel method. Cochrane Q test was used to assess heterogeneity; this was quantified using the (I^2^) statistic: no heterogeneity at 0%, low heterogeneity at 25%, moderate heterogeneity at 50%, and high/substantial heterogeneity at > 75%. To account for variability in heterogeneity, a random-effects model was used as recommended by the Cochrane Handbook for Systematic Reviews to increase robustness of our effect estimates. Forest plots were generated to visually represent outcomes reported by two or more studies with the same variables and units. The intervention arm describes the ‘extended’ group, and reference arm describes the ‘conventional’ or ‘limited’ group. Dichotomous variables were reported as odds ratio (OR) with 95% Confidence Interval (CI). OR was used as this meta-analysis involved case–control studies, which allows for an appropriate estimation of associations when the exact incidence cannot be reliably calculated. Continuous variables were reported as mean differences (MD) with 95% CI. Mean and SD were derived from median and range from original data using the Hozo et al. formula [15]. Categorical data was reported in the form of percentages or frequency. P value of < 0.05 was used as a cut-off for statistical significance.

## Results

### Demographics

**Fig. 3:**
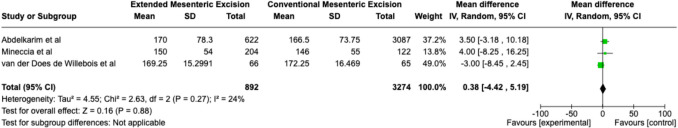
Forest plots of comparison of all outcomes (1) recurrence of disease, (2) re-operation, (3) postoperative morbidity, (4) Clavien-Dindo 3 + Complications, (5) intra-operative blood loss, (6) anastomotic leak, (7) length of stay, (8) Blood Transfusion Requirement, (9) Incidence of Postoperative Ileus, (10) intra-abdominal bleeding (11) re-admission rate (12) surgical site infection, (13) Postoperative Adjuvant or Prophylactic Therapy and (14) total operative duration. The solid squares denote the mean difference or odds ratio. The horizontal lines represent the 95% confidence intervals (CIs), and the diamond denotes the pooled effect size. M–H, Mantel–Haenszel test

Following screening of articles, five studies met our inclusion criteria: four were retrospective cohort studies [6, 7, 16, 17] and one RCT [5]. The summary of our search is provided in Fig. 1. A total of 4,358 patients were included in the meta-analysis, of which 993 patients formed the EMR group and 3,365 in the CMR group. The average age was 35.7 years (range 25–41) in the EMR group and 36.9 years (range 26–42) in the CMR group. The summary of patient demographics across the included studies is provided in Table 2. Primary and secondary outcomes are shown in Fig. 3.

### Primary outcomes

#### Disease recurrence

Four studies reported disease recurrence, either surgically or endoscopically diagnosed. No difference was observed between the two groups [OR 0.49 CI 0.21—1.16, P = 0.10]. There was significant heterogeneity between the studies [I^2^ = 76%, P = 0.006].

#### Re-operation rates

Three studies reported this outcome. No significant difference was observed between the two groups [OR 0.33 CI 0.06—1.65, P = 0.17]. There was significant heterogeneity between the studies [I^2^ = 88%, P = 0.0003].

### Secondary outcomes

#### Postoperative morbidity

Four studies reported post-operative morbidity. No difference was observed between the two groups [OR 1.01 CI 0.82—1.24, P = 0.95]. There was no heterogeneity detected between the studies [I^2^ = 0%, P = 0.81].

#### Clavien-Dindo III+ Complications

Two studies reported this outcome. No significant difference was observed between the two groups [OR 0.83 CI 0.5—1.38, P = 0.47]. There was no heterogeneity between the studies [I^2^ = 0%, P = 0.34].

#### Intraoperative haemorrhage

Two studies reported intra-operative blood loss. No difference was observed between the two groups [MD−18.71 CI−76.65—39.23), P = 0.53]. There was significant heterogeneity between the studies [I^2^ = 91%, P = 0.0001].

#### Anastomotic leak

Three studies reported this outcome. No difference was observed between the two groups [OR 0.98 CI 0.34—2.82, P = 0.97]. There was low heterogeneity between the included studies [I^2^ = 47%, P = 0.15].

#### Length of stay

Four studies reported this outcome. No difference was observed between the two groups [MD −0.06 CI −0.59—0.48, P = 0.83]. There was low heterogeneity between the studies [I^2^ = 42%, P = 0.16].

#### Blood transfusion requirement

Two studies reported this outcome. No difference was observed between the two groups [OR 1.16 CI 0.84—1.59, P = 0.36]. There was no between-study heterogeneity [I^2^ = 0%, P = 0.77].

#### Post-operative ileus

Two studies reported the occurrence of paralytic ileus post-operatively. No difference was observed between the two groups [OR 0.97 CI 0.27—3.50, P = 0.96]. There was low heterogeneity between the studies [I^2^ = 28%, P = 0.24].

#### Intra-abdominal bleeding

Two studies reported intra-abdominal haemorrhage. No difference was observed between the two groups [OR 0.85 CI 0.22—3.26, P = 0.81]. Cochran Q test revealed a low level of heterogeneity between the included studies [I^2^ = 30%, P = 0.23].

#### Re-admission rate

Two studies reported rates of re-admission. No significant difference was observed between the two groups [OR 0.65 CI 0.24—1.78, P = 0.40]. Cochran Q test revealed no heterogeneity between the included studies [I^2^ = 0%, P = 0.71].

#### Surgical site infections (SSI)

Two studies reported this outcome. No difference was observed between the two groups [OR 1.00 CI 0.77—1.30, P = 0.99]. There was no heterogeneity between the studies [I^2^ = 0%, P = 0.60].

#### Postoperative adjuvant or prophylactic therapy

Four studies reported this outcome. No difference was observed between the two groups [OR 0.90 CI 0.54—1.51, P = 0.69]. There was moderate heterogeneity between the studies [I^2^ = 52%, P = 0.10].

#### Total operative time

Three studies reported total operative time. No difference was observed between the two groups [MD 0.38 CI −4.42 – 5.19, P = 0.88]. There was low heterogeneity between the included studies [I^2^ = 24%, P = 0.27].

## Discussion

Our review found no significant difference in post-operative disease recurrence, re-operation rates or post-operative morbidity between patients undergoing extended or conventional/limited mesenteric excision. To our knowledge, this is the first review and meta-analysis of the available literature specifically comparing these two surgical strategies (EMR vs. CMR) in patients undergoing ileocolic resection for limited ileo-colonic CD.

The mesentery's role as a vital organ of the digestive, immune, and lymphatic systems has been extensively researched to gain a better understanding of mesenteropathies. Secondary mesenteropathy, which results in fat-wrapping, creeping, and thickening, is a standard pathogenesis and classical hallmark of CD [18]. The severity of inflammation and degree of fat wrapping are markers of disease severity [19].

There is also a suggestion that the aetiology of CD is probably primarily mesenteropathic in origin; much of the radiographic, endoscopic and surgical findings overlap between primary mesenteropathic inflammation and Crohn’s-related inflammatory process [20]. Therefore, mesenteric resection has been advocated in the surgical management of CD to achieve better clinical outcomes, although consensus on its significance remains controversial [21].

Approximately 25% of patients undergoing CD-related surgery develop post-operative complications [22]. Disease recurrence and re-operation rates remain high due to the extensive mesenteric involvement in CD, particularly granuloma formation and overwhelming activation of the mucosa-associated lymph tissue (MALT) [3, 23]. Therefore, the argument for retaining the mesentery—with a limited or sparing approach—is that surgical resection often occurs at delayed or advanced stages of disease, where severe mesenteric inflammation makes dissection and operating more difficult, increasing the risk of haemorrhage, infection and overall post-operative morbidity [20, 24, 25].

The influence and extent of mesenteric excision on disease recurrence and postoperative outcomes in ileocolic CD remains a matter of debate and discussion. To date, only five primary studies have attempted to answer this question, providing heterogeneous, non-consistent findings. The SPICY RCT [5] reported no difference in endoscopic recurrence six-months post-operatively between EMR and CMR in a cohort of 133 patients [*P* = 1.000]. There was also no reported significant difference in total operative time [*P* = 0.396], blood loss [P = 0.840], post-operative morbidity [P = 0.763] or the use of adjuvant therapy [*P* = 1.000] between the two groups.

Vaghiri et al. [26] recently published a meta-analysis of five studies and 4358 patients undergoing extended versus limited resection in Crohns Disease. The authors found a five-fold risk reduction in surgical recurrence with extended mesenteric excision [OR 4.94, P < 0.0001] compared to mesenteric preservation, with no significant differences between the two groups in terms of endoscopic recurrence, postoperative morbidity, and hospital stay. Although the authors’ work did not specifically address patients with only limited ileocolic CD, compared to our meta-analysis, their findings nonetheless support the conclusions of this systematic review and meta-analysis.

Coffey et al. [6] found that mesenteric inclusion was associated with a significantly lower disease recurrence rate and further re-operations [EMR 2.9%, CMR 40%, *P* = 0.003]. This is supported by their findings of increased correlation between mesenteric disease activity index and CD activity index [*P* < 0.0001]. Zhu et al. [7] observed significantly longer, recurrence-free periods of disease [*P* = 0.01] and, paradoxically, almost 40mls lower intraoperative blood loss in extended resection [*P* = 0.02].

The REMEDY [17] retrospective cohort study of 326 patients observed no difference in endoscopic disease recurrence postoperatively [*P* = 0.7], the use of adjuvant therapy [*P* = 0.3] or duration of surgery [*P* = 0.5] between the two cohorts. Their multivariate analysis found mesenteric resection to lack predictiveness of recurrence [HR 1.6, P = 0.09]. Abdelkarim et al. [16] conducted a retrospective review of the 30-day outcomes of 3,709 patients enrolled on the National Surgical Quality Improvement Program (NSQIP) database; no difference was observed in any intra- or post-operative complications.

Our meta-analysis found no difference in any of the analysed outcomes. The variable levels of heterogeneity, study demographics and low-level evidence of studies included may explain this; the retrospective nature of four studies automatically lends to randomisation bias. Similarly, some results did not delineate between prophylactic continuation of post-operative adjuvant therapy or if therapy was initiated for the first time. The significant heterogeneous population in Coffey et al. [6] and REMEDY [17] studies also introduce bias. Nonetheless, the no observed difference suggests a lack of observed benefit in undertaking extensive resections on postoperative complications, disease recurrence or the need for further ongoing medical therapy.

Randomised trials are taking place to evaluate these outcomes more robustly. Li et al. [9] have published a study protocol for an international, multi-centre RCT with preliminary data suggesting that extended mesenteric resection is safe, feasible and associated with lower post-operative recurrence. The MEErKAT trial [8] is also taking place to investigate those associations alongside investigating the Kono-S anastomotic technique. We look forward to the findings of these studies.

Whilst our study has shown no difference between the two cohorts, there are conceptual disadvantages associated with EMR. Contrary to historical concepts that refer to ‘extended’ excision when discussing ‘length’ of bowel, our study explored the concept of EMR in the context of ‘depth’ of mesenteric excision to its vascular origin. Therefore, an EMR may inevitably lead to an extended bowel resection, often well beyond the affected segment of bowel. Ultimately, EMR may well lead to absorption and nutritional problems that are difficult to quantify and compare. There will also be consequences for future operative management.

### Limitations

This review has some methodological limitations. Firstly, we only focused on the extent of mesenteric excision within the literature and excluded studies comparing anastomotic techniques; Holubar et al. [27] described their Kono-S anastomosis technique as a combined anastomosis with limited mesenteric excision, known as ‘Mesenteric Excision and Exclusion-MEE’. The SuPREMe-CD and KoCoRICCO studies were also excluded, although they provide high-level evidence [28, 29]. Though we recognise the value of these studies as they address techniques that may overlap with mesenteric resection, the focus of our review was solely to address the paucity of literature addressing the extent of mesenteric resection, as meta-analyses comparing anastomotic techniques already exist [30].

Secondly, four of five studies were retrospective cohort studies, which are low level in evidence with no propensity matching. However, the NOS and RoB scores for those studies suggest a low level of bias. Thirdly, the number of patients pooled undergoing ileocolic resection varied between 17% and 46.7% between the two cohorts, and not all studies provided data on histological results at the resection margin—which may influence adjuvant therapy.

Thirdly, whilst the general assessment of bias in our included studies was reported as low, the individual studies provided point estimates of varied outcome directions, which was particularly evident in anastomotic leak rates and intraoperative blood loss. These discrepancies could be best explained by differences in surgical techniques, surgeon experience, patient selection criteria, variability in definitions of clinical outcomes, and heterogeneity in perioperative management protocols across included studies. Therefore, we advocate for cautious and contextual interpretation of these results.

Finally, there was a high level of heterogeneity observed within our primary outcomes and variable degrees of heterogeneity in secondary outcomes, likely due to low population size and low study numbers. We adjusted accordingly by using the Mantel–Haenszel and random-effects models as suggested by the Cochrane Handbook [10], as the low small number of studies in this review precludes us from performing meta-regression or subgroup analysis.

Despite the limitations, our findings provide a strong basis for ongoing and future RCTs, such as the MEErKAT trial [8], and suggest the need for more robust research to explore the impact of surgical resection techniques on disease recurrence. Other factors, such as anastomotic configuration, extent of mesentery resection, the use of adjuvant and neoadjuvant therapy may play a role into decision making and future studies should investigate the influence of those factors on disease progression and patient outcomes. Nonetheless, the studies included in our review imply that those variables play a significant role, and would require further evaluation.

## Conclusion

The current mesenteric sparing approach remains safe and has no less benefit than extended resection. Accounting for limitations, the lack of inferiority in performing limited mesenteric resections supports the need for methodologically robust RCTs to compare short-, mid-, and long-term outcomes of patients undergoing both extents of resection to establish an optimal approach to managing the mesentery in limited ileo-colonic CD.

## Data Availability

No datasets were generated or analysed during the current study.
